# Editorial: New Insights in Skeletal Muscle Channelopathies - A Rapidly Expanding Field

**DOI:** 10.3389/fneur.2020.626772

**Published:** 2020-12-17

**Authors:** Lorenzo Maggi, Emma Matthews, Jean-François Desaphy

**Affiliations:** ^1^Neuroimmunology and Neuromuscular Diseases, Fondazione Istituto di Ricovero e Cura a Carattere Scientifico (IRCCS) Istituto Neurologico Carlo Besta, Milan, Italy; ^2^The Atkinson-Morley Neuromuscular Centre, St George's University Hospitals NHS Foundation Trust, London, United Kingdom; ^3^Department of Neuromuscular Diseases, Queen Square Institute of Neurology, UCL, London, United Kingdom; ^4^Department of Biomedical Sciences and Human Oncology, School of Medicine, University of Bari Aldo Moro, Policlinico, Bari, Italy

**Keywords:** SCN4A gene, CLCN1 gene, CACNA1S, myotonia, periodic paralysis, channelopathies

Skeletal muscle channelopathies (SMCs) are a heterogeneous group of rare genetic neuromuscular diseases resulting in long–term disabilities, posing a significant burden to patients, their families and National Health Care Services. SMCs are caused by mutations in genes encoding skeletal muscle ion channels that control muscle excitability, such as CLCN1, SCN4A, CACNA1S, and KCNJ2. The resultant effect of mutations on muscle excitability is to cause either episodic muscle weakness or myotonia. Based on these predominant clinical feature, SMCs are typically classified as non-dystrophic myotonias (NDMs) or periodic paralyzes (PPs). Given the episodic nature of clinical symptoms diagnosis can be challenging and requires a high degree of clinical suspicion. To date, only symptomatic treatments to reduce myotonia or frequency of paralytic attacks are available and there are no disease-modifying therapies. Although numerous drugs are proposed, data on their efficacy in SMCs consists mostly of case series, open-label and single-blind, controlled trials. The ability to perform randomized controlled trials to generate evidence based treatment approaches has been limited in part by disease rarity and the challenges of recruitment, although a few randomized controlled trials have recently been performed. Mexiletine is now generally considered the gold-standard treatment for NDM, following a phase 2 international randomized, placebo-controlled, crossover clinical trial and a series of N-of-1 trials of mexiletine vs. placebo. Mexiletine however, has reported side effects, mainly gastrointestinal, in a significant proportion of patients. Additionally, 10–30% of patients have a suboptimal or no response. In the periodic paralyzes carbonic anhydrase inhibitors have traditionally been used as the treatment of choice and a recent RCT confirmed the efficacy of dichlorphenamide in hypokalaemic PP. Treatment options are expanding however, and newer drugs have recently been reported as effective in SMC treatment, including flecainide, lamotrigine, and ranolazine. Recently, many achievements have increased complexity in the SMCs field, including the recognition of new phenotypes caused by mutations in *SCN4A* and *CACNA1S* genes, such as severe neonatal episodic laryngospasms, severe fetal hypokinesia, congenital myopathy, rhabdomyolysis, myalgia and exercise intolerance, congenital myasthenic syndrome, and sudden infant death syndrome. Conversely, new genes associated with PP (often with additional clinical features) have been reported, e.g., *ATP1A2, KCNJ5, RYR1, mATP6*, and *mATP8*. In addition, guidelines on clinical presentation and management of NDMs have been recently published by a panel of international experts (1).

Clinical studies reported in this Frontiers in Neurology special issue provide an up to date review of current knowledge of SMCs clinical phenotypes and management. They also highlight ongoing gaps in our knowledge e.g., clinically useful predictors of disease progression, and response to therapy, a lack of disease modifying therapies, inadequate randomized controlled trial evidence for many symptomatic treatments and the role of genotype specific drug efficacy in clinical practice.

Studies on specific patient populations are needed to better clarify SMCs genotype-phenotype correlations and disease epidemiology and to aid in the clinical interpretation of diagnostic genetic testing. In this regard, Orsini et al. described a cohort of patients with congenital myotonia caused by mutations in *CLCN1* gene, originating from a specific region in southern Italy, with a common set of mutations different from those reported in Italian patients in other regions. Maggi et al. reported clinical and molecular features of a large cohort of patients with *SCN4A* gene mutations, clinically ranging from PP to NDM and to neonatal disease, further supporting these phenotypes represent a continuum in the clinical spectrum. Further studies are warranted to investigate genetic and non-genetic modifiers of the phenotype.

Laboratory investigations of ion channelopathies are fundamental. First, they assist in confirming whether a new mutation is disease causing, especially in the case of a new or unusual clinical phenotype. Shi et al. described the effect of a sodium channel mutation associated with normokalemic periodic paralysis, while Elia et al. reported a new phenotype characterized by myopathy and joint and skeletal anomalies secondary to a severe myotonia. Functional studies shed light on the molecular mechanisms linking the genotype to the clinical phenotype. Altamura et al. described a ClC-1 chloride channel mutation linked to recessive myotonia congenita, which likely induces a protein misfolding resulting in non-functional channels. The molecular defects induced by ClC-1 channel mutations are reviewed by Jeng et al., showing a complexity of pathomechanisms in myotonia congenita. In this context, important hints can also be provided for by more physiological studies; For instance, the study by Leermakers et al. suggests that the ClC-1 channel may be regulated by ATP and operate as a sensor of skeletal muscle metabolic state, limiting muscle excitability when energy status is low. Regarding sodium channel myotonia, studies of Elia et al. and Pagliarani et al. highlighted the correlation between sodium channel functional defect and severity of the clinical phenotype. Yet, although many studies on the genotype/phenotype relationship have been already published, a number of issues are still unresolved, as reviewed by Morales and Pusch for myotonic syndromes. Importantly, such studies may help identifying possible treatment targets.

The treatment of SCMs remains largely focused on symptomatic improvement. Although RCT data is lacking for many therapies used in clinical practice significant data has been generated for the efficacy and safety of mexiletine in the short-term. Modoni et al. contribute to evidence that this efficacy is maintained in the long-term of clinical practice and provide further re-assurance of minimal cardiac side effects occurring in the SMCs. Meyer et al. illustrate that symptoms of weakness and pain are also prevalent in the NDMs and remind us that when considering the holistic treatment of patients these should not be ignored. It is clear that available therapies are not effective for or tolerated by all patients and newer targeted and disease modifying approaches are needed but Meyer et al. also provide data that morbidity may be impacted by an under-utilization of available anti-myotonics.

SMCs research is now moving toward the proposed interdisciplinary approach, possibly the only promising strategy to find efficient new treatments and a cure for these diseases. We are confident that this Frontiers in Neurology issue may represent a milestone in the field to make clear where we are and provide new paths.

## Author Contributions

LM, EM, and J-FD contributed to the Editorial. All authors contributed to the article and approved the submitted version.

## Conflict of Interest

The authors declare that the research was conducted in the absence of any commercial or financial relationships that could be construed as a potential conflict of interest.
